# Adaptation of Surface-Associated Bacteria to the Open Ocean: A Genomically Distinct Subpopulation of *Phaeobacter gallaeciensis* Colonizes Pacific Mesozooplankton

**DOI:** 10.3389/fmicb.2017.01659

**Published:** 2017-08-31

**Authors:** Heike M. Freese, Anika Methner, Jörg Overmann

**Affiliations:** Leibniz-Institut DSMZ-Deutsche Sammlung von Mikroorganismen und Zellkulturen Braunschweig, Germany

**Keywords:** *Phaeobacter gallaeciensis*, zooplankton, attached bacteria, bacterial adaptation, genome evolution, high-throughput cultivation

## Abstract

The marine *Roseobacter* group encompasses numerous species which occupy a large variety of ecological niches. However, members of the genus *Phaeobacter* are specifically adapted to a surface-associated lifestyle and have so far been found nearly exclusively in disjunct, man-made environments including shellfish and fish aquacultures, as well as harbors. Therefore, the possible natural habitats, dispersal and evolution of *Phaeobacter* spp. have largely remained obscure. Applying a high-throughput cultivation strategy along a longitudinal Pacific transect, the present study revealed for the first time a widespread natural occurrence of *Phaeobacter* in the marine pelagial. These bacteria were found to be specifically associated to mesoplankton where they constitute a small but detectable proportion of the bacterial community. The 16S rRNA gene sequences of 18 isolated strains were identical to that of *Phaeobacter gallaeciensis* DSM26640^T^ but sequences of internal transcribed spacer and selected genomes revealed that the strains form a distinct clade within *P. gallaeciensis*. The genomes of the Pacific and the aquaculture strains were highly conserved and had a fraction of the core genome of 89.6%, 80 synteny breakpoints, and differed 2.2% in their nucleotide sequences. Diversification likely occurred through neutral mutations. However, the Pacific strains exclusively contained two active Type I restriction modification systems which is commensurate with a reduced acquisition of mobile elements in the Pacific clade. The Pacific clade of *P. gallaeciensis* also acquired a second, homolog phosphonate transport system compared to all other *P. gallaeciensis*. Our data indicate that a previously unknown, distinct clade of *P. gallaeciensis* acquired a limited number of clade-specific genes that were relevant for its association with mesozooplankton and for colonization of the marine pelagial. The divergence of the Pacific clade most likely was driven by the adaptation to this novel ecological niche rather than by geographic isolation.

## Introduction

Bacteria of the genus *Phaeobacter* belong to the widespread and often abundant marine *Roseobacter* group (Buchan et al., 2005; Wagner-Döbler and Biebl, 2006; Simon et al., 2017). They are adapted to a surface associated lifestyle and are characterized by a high versatility of metabolic pathways (Martens et al., 2006; Dickschat et al., 2010; Newton et al., 2010; Thole et al., 2012). The cells attach tightly to abiotic and biotic surfaces, outcompeting other bacteria and can even invade established epiphytic communities (Rao et al., 2005, 2010; Frank et al., 2015). Colonization is accompanied by the production of the antibiotic tropodithietic acid (TDA) which inhibits a variety of bacteria including pathogenic vibrios (Brinkhoff et al., 2004; Porsby et al., 2008; Prado et al., 2009). The inhibitory activity may also involve antibacterial peptides encoded by hybrid non-ribosomal peptide synthetase/polyketide synthases (Ruiz-Ponte et al., 1999; Martens et al., 2007). Due to their antibiotic activity, members of the genus *Phaeobacter* exert a beneficial effect on different fish and shellfish and may act as probiotic in aquacultures (D’Alvise et al., 2012; Kesarcodi-Watson et al., 2012; Karim et al., 2013). However, *Phaeobacter* cells associated with senescent algae switch from a mutualistic to a parasitic lifestyle and produce algaecides (“roseobacticide”) leading to the lysis of algal cells (Seyedsayamdost et al., 2011b).

Despite their high metabolic versatility and contrary to other representatives of the *Roseobacter* group (Billerbeck et al., 2016; Sonnenschein et al., 2017), *Phaeobacter gallaeciensis, P. inhibens*, and *P. porticola* have so far been detected exclusively in different aquacultures (Porsby et al., 2008; Prado et al., 2009; Balcazar et al., 2010; Xue et al., 2016) or on sessile invertebrates and abiotic surface in harbors (Gram et al., 2015; Breider et al., 2017). In addition, only a few solitary isolates from macroalgae, seaweed, and intertidal mudflats at coastal shores of Australia, France and Germany have been reported (Rao et al., 2005; Martens et al., 2006; Penesyan et al., 2009; Doghri et al., 2015).

Two strains of *P. inhibens* which had been isolated from an aquaculture at the Atlantic Coast of north western Spain (strain DSM 17395) and from a macroalga near Sydney, Australia (strain 2.10, DSM 24588), respectively, had identical 16S rRNA sequences, a genome sequence identity of 97%, a very high percentage of shared genes (88–93%), and a high synteny of genomes and plasmids (Thole et al., 2012). Considering that *Phaeobacter* spp. were mostly detected on surfaces in man-made environments, the presence of highly similar genotypes in locations as far as 18,000 km apart suggest that rapid means of dispersal exist for these bacteria. Also, populations of *Phaeobacter* spp. might exist in other marine environments which so far have escaped detection by established molecular or cultivation-based approaches. Accordingly, *Phaeobacter* may also occur associated to phytoplankton or zooplankton in the open ocean. We hypothesize that attachment to and transport by planktonic eukaryotes link the *Phaeobacter* populations in distant coastal environments.

In metagenomics databases from the oceans like the Global Ocean Sampling, *Phaeobacter* spp. are not detectable. Although, short 16S rRNA gene sequence fragments related to *Phaeobacter* spp. are present in the Tara Ocean 16S rRNA sequence database^1^, they could not be unambiguously classified, as these short read sequences are also identical to several other *Roseobacter* group genera like *Sulfitobacter* and *Ruegeria* which are known to be globally distributed (Sonnenschein et al., 2017). Different *Phaeobacter* species and related genera have very low 16S rRNA gene sequence differences. In combination with their low abundance this currently precludes a reliable detection of these bacteria by culture-independent methods suitable for large sample sets. We therefore conducted a systematic study to detect *Phaeobacter* in the open Pacific Ocean along a longitudinal transect from New Zealand to Alaska employing advanced high-throughput enrichment methods with improved growth media. Subsequent comparative genomics of selected *Phaeobacter* isolates revealed a distinct subpopulation of *P. gallaeciensis* with particular adaptations. These findings contribute to our understanding of the evolution and dispersal of marine surface-associated bacteria.

## Materials and Methods

### Sampling and Sample Preparation

Plankton samples were collected during cruise SO248 of the RV Sonne in May 2016 at 12 open water stations in the Pacific along a longitudinal transect between Auckland (New Zealand) and Dutch Harbor (Alaska) (**Table 1**). For sampling, a 3.2 m long Bongo net with an opening diameter of 61 cm and mesh sizes of 100 or 300 μm was employed. Vertical tows were conducted from 150 m water depth to the surface with a constant velocity of 0.2 m s^-1^ and horizontal hauls were done at the water surface for 45 min at a speed of 1.5–2 knots. Two ml biovolume of concentrated plankton was homogenized with a glass tissue grinder (Kimble Chase Gerresheimer, United States) and diluted with artificial sea water (ASW; Bruns et al., 2003) supplemented with 10 mM HEPES, pH 7.6. Samples from the supernatant of the plankton sample were used for parallel inoculations to investigate the bacteria which were not firmly attached to the zooplankton. A workflow scheme of the combined methods is depicted in **Supplementary Figure S1**.

**Table 1:** Characteristics of samples and sampling depths. Temperature and salinity were recorded by a CTD system and taken from Badewien et al. (2016).

Station	Date time UTC	Local	Latitude	Longitude	Temperature (°C)		Salinity (PSU)		Samples	Cultivation
		time			Surface	150 m	Surface	150 m	^∗^	
SO248_1	02.05.2016 20:51	08:51	29° 59.999′ S	177° 0.002′ E	23.5	17.9	35.9	35.8	100 and 300 μm vertical, supernatant	High-throughput liquid, agar plates
SO248_4	08.05.2016 00:47	13:47	10° 20,001′ S	176° 29,933′ W	30.1	23.7	34.7	36.2	300 μm horizontal	Agar plates
SO248_6	12.05.2016 05:19	18:19	0° 0,005′ N	180° 0,000′ E	29.5	19.7	35.2	35.3	300 μm horizontal	Agar plates
SO248_8	16.05.2016 00:34	13:34	11° 0,008′ N	179° 0,331′ E	27.8	16.2	34.6	34.7	300 μm horizontal	Agar plates
SO248_10	18.05.2016 23:52	13:52	22° 0,047′ N	178° 18,985′ E	25.2	18.0	35.5	35.0	300 μm horizontal	Agar plates
SO248_12	21.05.2016 20:08	10:08	33° 59.912′ N	177° 20.018′ E	15.8	15.0	34.8	34.7	300 μm horizontal, supernatant	High-throughput liquid
SO248_13	23.05.2016 08:18	22:18	39° 58.226′ N	177° 19.611′ E	11.9	9.3	34.3	34.2	100 μm vertical, 300 μm horizontal, supernatant	High-throughput liquid
SO248_14	24.05.2016 14:09	04:09	45° 0.002′ N	178° 44.990′ E	5.8	5.4	33.3	33.5	100 μm vertical, supernatant	High-throughput liquid
SO248_16	26.05.2016 23:31	13:31	49° 59.462′ N	179° 31.444′ E	4.8	3.6	33.1	33.9	300 μm vertical, supernatant	High-throughput liquid
SO248_17	29.05.2016 03:30	17:30	54° 0.081′ N	179° 34.889′ E	4.6	3.3	33	33.5	300 μm horizontal, supernatant	High-throughput liquid
SO248_18	30.05.2016 04:50	19:50	56° 59.189′ N	179° 37.476′ E	4.2	3.8	33.2	33.5	100 μm vertical, supernatant	High-throughput liquid
SO248_19	31.05.2016 01:37	16:37	58° 54.472′ N	178° 55.749′ W	5.4	3.7	32.9	33.2	100 μm vertical, supernatant	High-throughput liquid

^∗^*Underlined samples yielded Phaeobacter.*

### Enrichment, Isolation, and Screening Strategies

High-throughput liquid serial dilutions were performed on board in 96 deepwell plates (2 ml MASTERBLOCK^®^, Greiner Bio-One GmbH) with 48–128 inoculated wells per dilution (10^-4^ to 10^-10^) and sample. Two different media were successfully employed to grow *Phaeobacter*. Medium HD contained some complex organic substrates at low concentrations and based on ASW supplemented with 0.6 mM glucose, 0.25 g l^-1^ yeast extract, 0.5 g l^-1^ peptone, 20 mg l^-1^ cycloheximide, 0.2 mM ferric citrate, 1 ml l^-1^ vitamin solution (Karsten and Drake, 1995), and 1 ml l^-1^ trace element solution SL 10 (Widdel et al., 1983). Medium AM was more alkaline (pH 9) and based on ASW after Martens et al. (2006) but without silicate and with 10 mM CHES, 0.2 mM ferric citrate, 1 ml l^-1^ selenite-tungstate solution (Tschech and Pfennig, 1984), supplemented with 0.5 mM glucose, 0.5 mM D-alanine, and 0.2 mM Tween^®^ 80. To test isolation success on solidified media, plankton homogenate was streaked onto Difco marine broth Bacto agar (BD) in dilutions from 10^-4^ to 10^-8^ in duplicates. Deepwell plates and agar plates were incubated at 15°C while one parallel of each dilution of solid medium was incubated at *in situ* temperature for at least 10 days to test for temperature effects.

To detect *Phaeobacter* and closely related genera, cultures were screened first on board and later in our home lab via PCR using the specific forward primer PHA-16S-129f (5′-AAC GTG CCC TTC TCT AAG G-3′; Gram et al., 2015) and the universal reverse primer 907r (5′-CCG TCA ATT CMT TTG AGT TT-3′; Lane, 1991) (details see Supplementary Material). Positive cultures were sequenced and identified according to the NCBI type material database (Federhen, 2015). Most probable numbers (MPNs) of *Phaeobacter* were calculated after Jarvis et al. (2010). Strains of *P. gallaeciensis* were isolated from positive enrichments by streaking on solid media.

### Sequencing of the 16S rRNA Gene and the Internal Transcribed Spacer Region

To sequence the complete 16S rRNA gene and the downstream internal transcribed spacer (ITS) region, isolates were grown in marine broth, harvested by centrifugation and DNA was extracted by the Qiagen Blood & Tissue Kit. Amplification products were generated with primers 27f (5′-AGA GTT TGA TCM TGG CTC AG-3′; Lane, 1991) and 23S-130r (5′-GGG TTB CCC CAT TCR G-3′; Fisher and Triplett, 1999) and sequenced by Sanger sequencing. ITS sequences were aligned using ClustalW implemented in MEGA6 (Tamura et al., 2013) and manually curated. The phylogenetic tree was calculated employing the best fit option of the Maximum Likelihood method (Kimura 2-parameter model with gamma distribution, complete gap deletion) in MEGA6.

### Genome Sequencing, Assembly, and Annotation

Based on the results of the phylogenetic analysis, two strains originating from distant sampling locations were chosen for genome analysis. Genomic DNA was extracted with the JETFLEX Genomic DNA Purification Kit (Genomed). SMRT sequencing was carried out on the PacBio *RSII* (Pacific Biosciences, Menlo Park, CA, United States) using the P6 chemistry (details see Supplementary Material). PacBio reads were assembled *de novo* in the SMRT Portal 2.3.0 and were corrected by paired-end Illumina reads, which were sequenced on the MiSeq (PE150), using the Burrows-Wheeler Aligner (Li and Durbin, 2009) and the CLC Genomics Workbench 7.0.1. The final assembly was circularized and adjusted to the replication system as start point^2^. Genome sequences were automatically annotated using Prokka 1.8 (Seemann, 2014) and were deposited in the NCBI GenBank (accession numbers: CP021040–CP021052). Information for all other *Phaeobacter* strains which were used for genomic comparisons are given in Supplementary Table S1.

### Genome Comparisons

Polymorphic genome sites in the *Phaeobacter* genus were extracted from a core genome alignment using Parsnp and Gingr (Treangen et al., 2014). A phylogenetic network was calculated from the resulting matrix which contained 68,858 characters with the NeighborNet algorithm in SplitsTree 4.13.1 (Huson and Bryant, 2006). Phylogenetic distances of *P. gallaeciensis* were inferred from pairwise comparisons of complete genome sequences via the GGDC 2.1 web service (formula 2; Meier-Kolthoff et al., 2013). A BIONJ tree was calculated with the R package ape and rooted at midpoint (Gascuel, 1997; Paradis et al., 2004). Whole chromosome alignments of *P. gallaeciensis* were obtained with Mauve (Darling et al., 2010), sequence similarity between genomes estimated by BLAST (Altschul et al., 1990) and plotted together with the R package genoPlotR (Guy et al., 2010). Nucleotide diversity between Pacific and the other strains was calculated for all aligned ortholog genes along the genome with the R package PopGenome (Pfeifer et al., 2014). Prophages were identified with PHASTER (Arndt et al., 2016) and classified by Virfam (Lopes et al., 2014). Genomic islands were predicted with the IslandViewer web server (Dhillon et al., 2013). Mobile elements of the other *P. gallaeciensis* had been identified previously (Freese et al., submitted) and were included in the analysis. Orthologs of *P. gallaeciensis* were inferred via proteinortho (Lechner et al., 2011) and the core genome was defined as orthologs present in all strains. Subsequently, clade-specific orthologs were identified and their KEGG orthologies (ko identifiers) were determined using KAAS (Moriya et al., 2007) using a threshold of 30%. Methylation motifs were detected using the SMRT Portal with the RS Modification and Motif Analysis and were identified using REBASE (Roberts et al., 2015). Methylase sequence homologs for the hits were identified using BLAST.

## Results

**FIGURE 1:**
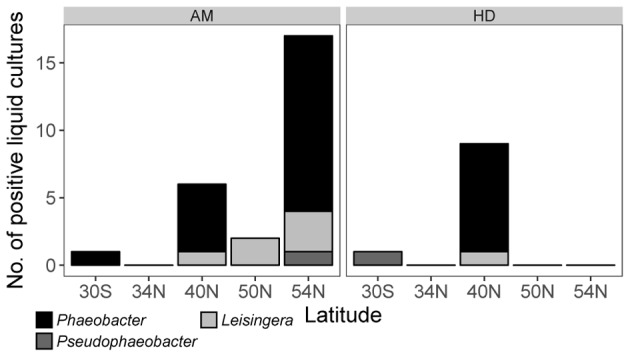
Number of positive enrichments of *Phaeobacter* and phylogenetically related genera obtained from zooplankton > 300 μm collected along a longitudinal transect in the Pacific (respective Latitudes of sampling sites are given) in defined medium AM or complex medium HD.

For the first time, *Phaeobacter* spp. were successfully enriched from the open ocean by employing our high-throughput liquid cultivation. Enrichments were obtained from very distantly located samples taken in the South Pacific (30°S), North Pacific (40°N), and in the Bering Sea (54°N) (**Figure 1**) where surface water temperatures differed between 23.5 and 4.6°C (**Table 1**). The observations that representatives of the phylogenetically related genera *Pseudophaeobacter* and *Leisingera* were also enriched and that total MPNs exceeded those of *Phaeobacter* by 1–2 orders of magnitude (**Table 2**) indicate that both types of liquid media were not highly selective for *Phaeobacter*. Cultivation attempts on marine broth agar failed for samples from the open Pacific (**Table 1**) even when liquid enrichments from the same station (SO248_1) yielded *Phaeobacter*. Most notably, *Phaeobacter* were exclusively enriched from size fractions >300 μm which consists mostly of larger zooplankton organisms. In contrast neither the supernatant nor the size fraction >100 μm yielded enrichments of *Phaeobacter* even at stations SO248_1 and SO248_13 where both size fractions were tested in parallel.

**Table 2:** Most probable numbers (MPNs) of *Phaeobacter* per ml plankton biovolume.

Station	Medium	*Phaeobacter*			Total numbers		
		MPN ml^-1^	95% confidence limits		MPN ml^-1^	95% confidence limits	
30°S	HD	0			1.5⋅10^6^	1.1⋅10^6^	1.9⋅10^6^
30°S	AM	1.3⋅10^3^	1.7⋅10^2^	9.5⋅10^3^	7.4⋅10^5^	5.9⋅10^5^	9.3⋅10^5^
34°N	HD	0			6.4⋅10^5^	4.4⋅10^5^	9.3⋅10^5^
34°N	AM	0			2.0⋅10^5^	1.5⋅10^5^	2.5⋅10^5^
40°N	HD	1.2⋅10^5^	5.7⋅10^4^	2.3⋅10^5^	1.1⋅10^7^	8.7⋅10^6^	1.4⋅10^7^
40°N	AM	7.2⋅10^4^	2.9⋅10^4^	1.8⋅10^5^	6.3⋅10^6^	4.8⋅10^6^	8.1⋅10^6^
50°N	HD	0			6.0⋅10^6^	4.9⋅10^6^	7.4⋅10^6^
50°N	AM	0			6.5⋅10^5^	5.5⋅10^5^	7.7⋅10^5^
54°N	HD	0			3.4⋅10^6^	2.7⋅10^6^	4.4⋅10^6^
54°N	AM	1.7⋅10^4^	1.0⋅10^4^	3.0⋅10^4^	2.3⋅10^5^	1.9⋅10^5^	2.9⋅10^5^

The specific MPNs calculated from the results of the liquid dilution series revealed highest *Phaeobacter* abundances of 7.2⋅10^4^ to 1.2⋅10^5^ cells (ml plankton biovolume)^-1^ in the North Pacific (**Table 2**). In the warmer Southern Pacific, the *Phaeobacter* abundance was 2 orders of magnitude lower since only one well of the liquid dilution series proved to be positive (**Figure 1**).

**FIGURE 2:**
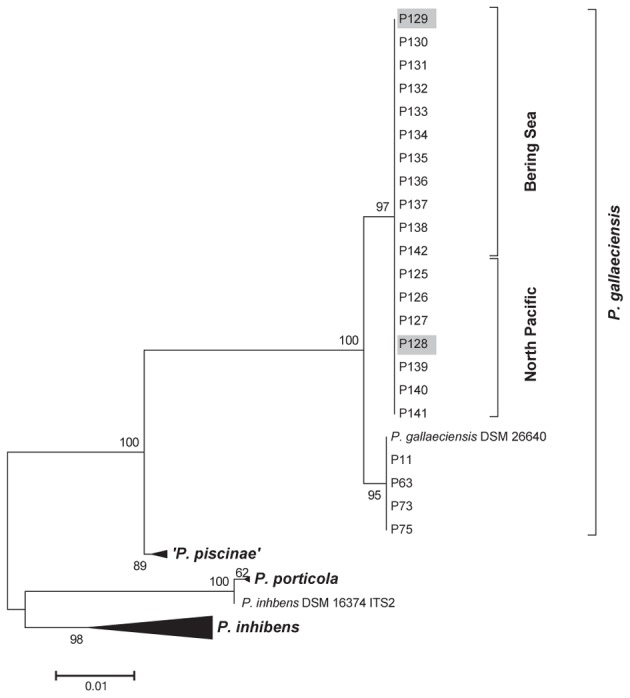
Maximum likelihood phylogenetic tree of *Phaeobacter* strains based on ITS sequences. For comparison, *Phaeobacter* strains representing different ITS lineages identified previously (Breider et al., 2017; Freese et al., submitted) were chosen. The tree based on 767 aligned nucleotide positions. Numbers adjacent to branches give percentage of support from 1000 bootstrap replicates. Strains chosen for genome sequencing are marked.

A total of 18 strains were isolated from the samples collected in the North Pacific and the Bering Sea. The 16S rRNA gene sequences of all isolates were identical to the sequence of the *P. gallaeciensis* type strain DSM 26640^T^ and to all other isolates of *P. gallaeciensis* which are available to date. The ITS sequences of all Pacific strains were also identical but clustered separately from the other *P. gallaeciensis* sequences due to five polymorphic sites (**Figure 2**). Based on the results of the ITS analysis, two strains isolated from locations 1500 km apart in the North Pacific (strain P128) and the Bering Sea (strain P129) and which had been enriched in two different media were chosen for genome sequencing. Their high quality, closed genome sequences were generated and compared to the five existing genome sequences of *P. gallaeciensis* strains originating from aquacultures in Spain and France (Supplementary Table S1).

**FIGURE 3:**
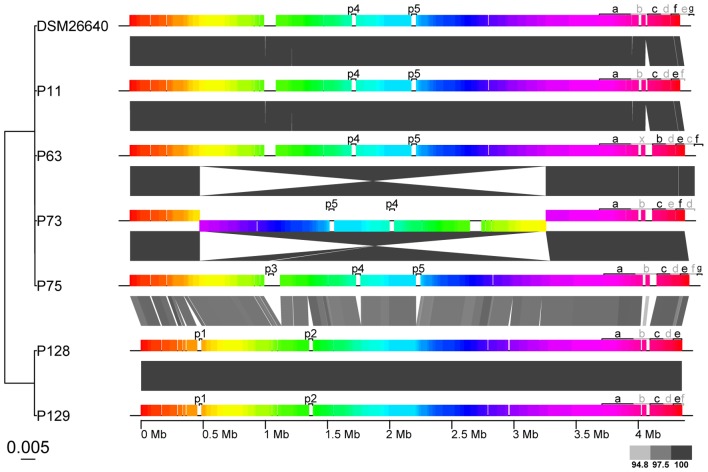
Synteny and sequence similarity of the 7 *Phaeobacter gallaeciensis* strains. The Mauve alignment depicts large collinear, and presumably homologous blocks as colored rectangles. Collinear blocks cover 87.2–92.4% of the whole genomes. Gray scale of wedges between the genomes indicates the level of sequence identity (%) between genomic blocks. The genome phylogeny on the left was inferred from pairwise genome to genome nucleotide sequence distances (GGDC) using BIONJ and the scale gives the genomic distance. Prophages (labeled p), and plasmids (labeled according to their names, for instance “a” corresponding to pP11_a or pP128_a) are also depicted.

**FIGURE 4:**
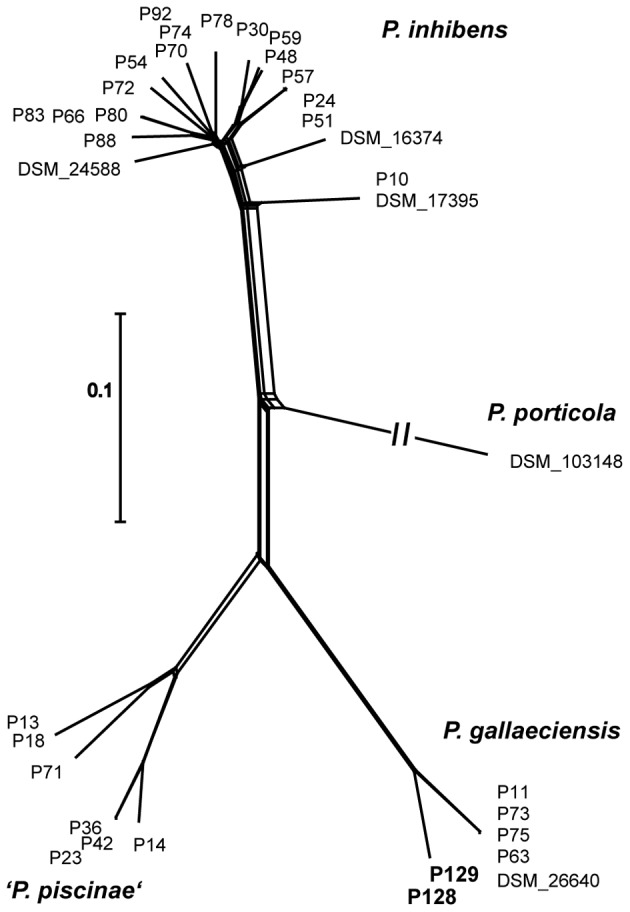
Phylogenetic network of 35 *Phaeobacter* strains inferred by NeighborNet analysis from 68858 core-genome SNPs estimated by Parsnp. Scale bar, 0.1 changes per polymorphic site. The long branch to *P. porticola* is depicted incompletely and Pacific strains are highlighted in bold.

The characteristics of the *P. gallaeciensis* genomes are summarized in **Table 3**. They share a large core genome (median, 89.6%), a high nucleotide similarity (median whole genome nucleotide similarity estimated as digital DNA:DNA hybridisation, 100%) and are highly syntenous with not more than 80 breakpoints (**Figure 3**). A considerable number of synteny breaks (31.5%) resulted from small sequence length variations (median 36 bp) in intergenic regions and 39.7% of the breaks occurred due to differences in gene content. The two isolates from the open Pacific were nearly identical in their genome sequence and differed by just 17 SNPs. Of these SNPs, 6 were located in intergenic regions whereas 11 were located in single core orthologs (nine non-synonymous, two synonymous). The two genomes of the Pacific strains differed by only 15 genes which are located on a small, 15-kb plasmid present in P129 but not P128 (labeled f in **Figure 3**). This plasmid carried a replication system and also a restriction system. Among the total of seven *P. gallaeciensis* strains, 84,374 SNPs were detected which were nearly homogenously distributed over the whole genome (**Supplementary Figure S2**). The Pacific strains differed by 83,953 SNPs from the five aquaculture *P. gallaeciensis* strains whereas much fewer differences were detected among the latter (405 SNPs). Phylogenetic network analysis confirmed that the Pacific isolates constitute a separate clade within *P. gallaeciensis* with no indication of recombination between the clades (**Figure 4**). Between the two clades, the average nucleotide similarity of homolog blocks still amounted to 97.8%. A higher sequence divergence was only detected for the RepABC-5 plasmid which also differed in gene content (**Figure 3**; plasmid labeled ‘b’ or ‘x’ have only 94.7% sequence similarity).

**Table 3:** Characteristics of *Phaeobacter gallaeciensis* genomes.

	Strains	Size	No. of	mol%	Genome	DDH	No. of	% core	% clade	% unique	Clade
		(Mb)	plasmids	G+C	sequence	(%)	genes	genes	core	genes	specific
					dissimilarity (%)						genes
Median	7	4.54	6	59.49	0.01	100.0	4339	89.6	na^∗^	0.02	na
Minimum	7	4.35	5	59.44	0	81.2	4098	87.7	na	0.00	na
Maximum	7	4.61	7	59.62	2.19	100.0	4432	94.7	na	1.22	na
Median pacific	2	4.36	5.5	59.61	0	100.0	4106	94.5	99.8	0.06	170
Median other strains	5	4.54	7	59.46	0.01	100.0	4359	89.1	96.1	0.02	316

^∗^*na, not applicable*.

*DDH, whole genome nucleotide similarity estimated as digital DNA:DNA hybridisation*.

**FIGURE 5:**
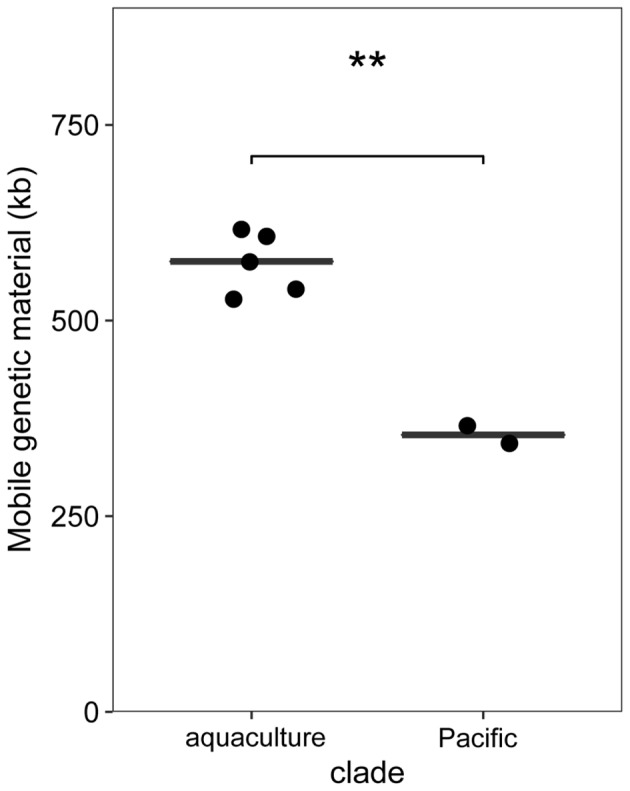
Differences in the summed sequences of mobile elements (plasmids, phages, genomic islands) between *P. gallaeciensis* clades. Horizontal lines show median and ^∗∗^ denotes *t*-test with *p* < 0.002.

*Phaeobacter gallaeciensis* strains from the Pacific contained significantly fewer mobile genetic elements than the five strains from aquacultures (*t*-test *p* < 0.002). Plasmids, prophages, and genomic islands together amounted 10.6% (464 kb) and 14.4% (653 kb) of the genomes, respectively (**Figure 5**). Significant differences were also apparent when only the mobile elements located in the chromosomes are considered (216 and 292 kb, *t*-test *p* < 0.001). While the overall number of prophages did not differ between the two *P. gallaeciensis* clades, different taxa of prophages were present. Both Pacific strains contained two Myoviridae (labeled p1 and p2; **Figure 3**) whereas all members of the aquaculture clade harbored a representative each of the Siphoviridae (p4) and of the Podoviridae (p5). One aquaculture strain, P75, in addition contained a member of the Myoviridae (p3), which was totally dissimilar on nucleotide and protein level to the Myoviridae detected in the Pacific *P. gallaeciensis*. Interestingly, this latter prophage occurred in a large 100 kb-region which contains several transferred genetic elements but is completely lacking in the Pacific clade (**Figure 3**). At this particular position, other *Phaeobacter* species also contained different mobile elements (Freese et al., submitted) which may indicate a selective loss in the Pacific clade after its divergence.

All *P. gallaeciensis* strains contained the TDA gene cluster and genes involved in surface attachment and biofilm formation (like motility, chemotaxis, polysaccharide biosynthesis and transport, rfb genes). On the other hand, comparative analysis of the functional gene content identified 170 and 316 clade-specific orthologs in the Pacific and aquaculture strains of *P. gallaeciensis*, respectively (**Table 3**). Functional assignments were possible for a limited number of these orthologs (**Table 4**). In the aquaculture strains these innovative orthologs often represented functional orphans, i.e., they occur isolated in metabolic pathways like in the terpenoid metabolism, or as part of xenobiotics biodegradation or host infections. The aquaculture strains may also contain few clade-specific genes coding for alternative reactions in carbohydrate or amino acid metabolism like a glutamate dehydrogenase (K00262), which may be involved in fixation or release of ammonia.

**Table 4:** Specific functional orthologs of *Phaeobacter gallaeciensis* clades.

Functional category	Pacific clade	Aquaculture
		clade
Amino acid metabolism		K01580, K00262, K01697
Carbohydrate metabolism	K00853, K01214	K01569, K00177, K05857, K03737
Catalase		K03781 (3x)
Cell growth and death		K03386, K03364
Energy metabolism		K02147
Glycan biosynthesis and metabolism		K05365
Host infection		K04646, K15352, K05692
Metabolism of cofactors and vitamins	K02549, K02551	
Metabolism of terpenoids and polyketides		K11731, K00587
Nervous system		K12460
Nucleotide metabolism		K02319
Replication and repair	K07466	K06223
Restriction and modification system (R-M system)	K03427 (2x), K01154 (2x), K01153 (2x), K07448	
Signal transduction	K04854, K12293	
Toxin–antitoxin system (TA system)		K07154
Transcription	K03142	
Translation	K14564	K14293, K03243
Transport and catabolism		K05940
Xenobiotics biodegradation and metabolism		K10221, K16046, K00517

In contrast to the aquaculture strains, the major innovative function of the Pacific strains is a complete Type I restriction/modification system (K03427, K01154, K01153) which occurred twice (i.e., PhaeoP128_00141 to 00144 and PhaeoP128_00349 to 00351). A detailed analysis of methylation patterns revealed two previously unknown Type I restriction motifs which occur exclusively in the Pacific clade (**A**GCN_6_GTCY and AGC**A**N_8_TTYG; **Table 5**). On average, 99.1% (98.2–99.7%) of these two motifs were found to be methylated in the genomes of the Pacific strains, indicating that the Type I restriction modification systems are active. All *P. gallaeciensis* strains encode an identical, active Type II restriction system (modification methylase BabI, ribonuclease HII). Some further methylated motifs of restrictions systems were detected but the corresponding genes for methylases could not be identified. Remarkably, the Pacific clade of *P. gallaeciensis* also acquired a second, homolog phosphonate transport system (PhnCDE) in addition to the phosphonate transport system/C–P lyase enzyme complex (PhnGHIJKLM) present in all other *P. gallaeciensis*.

**Table 5:** Presence of restriction system motifs in *Phaeobacter gallaeciensis* strains.

	R-M characteristics			Strain						
Motif^∗^	Modification	Type	Sub type	P128	P129	DSM 26640	P11	P63	P73	P75
**A**GCN_6_GTCY	m6_A_	I	nd	**+**	**+**	na	**-**	**-**	**-**	**-**
AGC**A**N_8_TTYG	m6_A_	I	nd	**+**	**+**	na	**-**	**-**	**-**	**-**
G**A**NTC	m6_A_	II	beta	**+**	**+**	na	**+**	**+**	**+**	**+**
TTCG**A**G	m6_A_	II	G,S, gamma	**-**	**-**	na	**+**	**+**	**+**	**+**
C**A**CN_3_GTG	m6_A_	II	gamma	**-**	**+**	na	**-**	**-**	**-**	**-**
CG**A**TCG	m6_A_	II	nd	**-**	**-**	na	**+**	**+**	**+**	**+**
GGCG**A**G	m6_A_		nd	**+**	**+**	na	**-**	**-**	**-**	**-**
CGCRG**A**C	m6_A_		nd	**-**	**-**	na	**-**	**+**	**+**	**-**

^∗^*Methylated position within the motif is highlighted in bold and underscored when occurring on the complementary strand*.

*na: genome of strain DSM 26640 was not done by SMRT sequencing therefore information on methylation pattern is not available*.

## Discussion

*Phaeobacter* spp. were detected in the >300 μm size plankton fraction which contained mostly mesozooplankton except for station SO248_12 at 34°N where samples were dominated by radiolarian macrocolonies. In contrast, *Phaeobacter* spp. was neither detected in the free-living bacterioplankton nor in the plankton fraction >100 μm. Due to the overall larger biovolume of the smaller plankton, the mesozooplankton (>300 μm) was highly diluted in the size fraction >100 μm which likely is the reason for the lack of *Phaeobacter* in these samples. Our results reveal that *Phaeobacter* occurs associated with zooplankton in the open ocean but in contrast to our initial expectations we found no evidence for their association with phytoplankton. Although *Phaeobacter* was previously shown to colonize macroalgae and laboratory cultures of dinoflagellates (Rao et al., 2005; Frank et al., 2015) a symbiotic interaction with algae was so far only demonstrated for the coccolithophore *E. huxleyi* (Seyedsayamdost et al., 2011a,b). In addition to zooplankton, *Phaeobacter* spp. may therefore also be specifically associated with some phytoplankton taxa which were not prevalent during the time of our cruise (e.g., Alvain et al., 2008).

Whereas, little information is available on the bacterial colonization of mesozooplankton in the open ocean, the total abundance of bacteria associated with estuarine or North Sea zooplankton was found to range between 1.2⋅10^8^ and 3.6⋅10^12^ (ml zooplankton biovolume)^-1^ (Møller et al., 2007; Bickel and Tang, 2014). Based on these total bacterial cell numbers, the abundance of *Phaeobacter* spp. detected by our cultivation-based approach would amount to ≤0.1% of all zooplankton-associated bacteria. By comparison, marine vibrios constitute between 1 and 26% of the associated bacterial community (Heidelberg et al., 2002). The low abundance of *Phaeobacter* spp. on Pacific mesozooplankton resembles the low percentage of *P. inhibens* sequence reads determined in biofilms colonizing inert surface or marine animals in harbors (Gram et al., 2015) and thus may constitute a typical feature of the genus. As *Phaeobacter* can exert antibacterial activities against marine pathogens and prevent biofouling at low cell densities (Rao et al., 2007) they are likely of ecological relevance for their hosts even at the low abundances deduced in the present study.

So far, *Phaeobacter* spp. have neither been found associated with zooplankton in coastal and shelf areas (Møller et al., 2007; Tang et al., 2009) or in oligotrophic open ocean (Shoemaker and Moisander, 2015). Elevated temperatures (above ∼18°C) were considered as a main factor determining the occurrence of *Phaeobacter* in harbors (Gram et al., 2015) but we could not confirm this for the open ocean. It remains to be investigated if *Phaeobacter* is preferentially associated with specific zooplankton taxa and changes in abundance according to the seasonal abundance of their hosts as was described for other bacterial groups (Turner et al., 2009; Tang et al., 2010). However, considering that zooplankton-associated bacteria typically constitute only <0.1–0.3% of all water column bacteria in the marine environment (Møller et al., 2007; Bickel and Tang, 2014), *Phaeobacter* spp. must represent a very rare bacterial group in marine water samples with abundances lower (<10^-3^ %) than the detection limit of many studies employing next generation sequencing (0.64%; Pochon et al., 2013). Commensurate with these results the metagenomes of a large number of oceanic samples typically do not contain any 16S rRNA gene sequence specific for *Phaeobacter* spp. In contrast, our cultivation-based high-throughput approach in optimized media enabled us to recover representative genotypes despite their low *in situ* abundance and to study their specific features.

Even by cultivation-based methods, *Phaeobacter* spp. have so far only been detected in anthropogenic habitats like aquacultures and harbors (Hjelm et al., 2004; Prado et al., 2009; Gram et al., 2015; Xue et al., 2016) or were sporadically isolated from shoreline samples (Rao et al., 2005; Martens et al., 2006; Penesyan et al., 2009). In general solid marine broth agar media have been employed to isolate *Phaeobacter*. Although we used Pacific samples for which *in situ* temperatures were close to the temperature optimum of growth of *P. gallaeciensis* DSM 26640^T^ and *P. inhibens* DSMZ 16374^T^ (23–27 and 27–29°C, respectively; Ruiz-Ponte et al., 1998; Martens et al., 2006), our attempts to isolate *Phaeobacter* on solid media were not successful. This suggests that agar media might be less suitable for recovering *Phaeobacter* from the open ocean. The *Phaeobacter* strains obtained in the present study employing optimized liquid enrichment strategies represent the first isolates from the open ocean environment. These genotypes constitute a phylogenomically separate subclade and hence do not represent known variants that link the distant coastal populations across the globe. Therefore their genomes were investigated for potential mechanisms of adaption to the open ocean habitat and to the inferred association to mesozooplankton.

All known genetic elements characterizing the surface- and host-associated lifestyle of *Phaeobacter* (Thole et al., 2012; Frank et al., 2015) are generally present in both *P. gallaeciensis* clades. The most prominent genomic features of the Pacific strains are their two complete, exclusive, and active Type I restriction modification systems which act as phage defense mechanism (Stern and Sorek, 2011; Loenen et al., 2014). Unlike their aquaculture counterparts, the Pacific strains only contained one type of prophage despite the high abundance and diversity of bacteriophages present in the ocean (Breitbart, 2012), suggesting an effective protection of the host against a broader range of bacteriophages. These restriction systems also are likely to prevent the horizontal transfer of foreign DNA through gene transfer agents (Stern and Sorek, 2011; Lang et al., 2012) which represent a major mechanism of gene acquisition of the genus *Phaeobacter* (Freese et al. submitted). This may explain the lower number and functional diversity of mobile elements observed in the Pacific strains of *P. gallaeciensis* compared to their aquaculture relatives. In addition, the slight reduction of the genome size of the Pacific strains by 0.18 Mb maybe related to an incipient genome streamlining as a consequence of nutrient limitation (Giovannoni et al., 2014; Luo and Moran, 2014). In this context it is remarkable that the genotypes of the Pacific *P. gallaeciensis* clade not only encode the complete phosphonate transport and degradation complex (cf. Villarreal-Chiu et al., 2012) like the aquaculture strains but in addition acquired and maintain a second set of phosphonate transporter (PhnCDE) despite their genome reduction. Phosphonates are particular prominent among marine invertebrate (Quin, 2000). Phosphonates also constitute a third of the dissolved organic phosphorus in oceanic waters and marine bacteria from the *Roseobacter* group have been shown to be capable of utilizing these compounds (Martinez et al., 2010). Pacific genotypes of *P. gallaeciensis* may therefore be particularly adapted to this alternative source of phosphorus.

Zooplankton surfaces are colonized by distinct bacterial communities which vary between zooplankton species and body regions (Tang et al., 2010). It has yet to be determined whether *P. gallaeciensis* can preferentially colonize the body surface or the intestinal tract of mesozooplankton or is also present on other organisms and particles >300 μm. Zooplankton functions as microbial hotspots since it provides a nutrient-richer environment than the surrounding water (Tang et al., 2010). A possible reciprocal advantage of *P. gallaeciensis* for the host could be related to the formation of TDA and other antibiotics that might affect the success of other bacteria to colonize zooplankton.

The genomes of the two Pacific strains were highly similar (97.8% nucleotide similarity; fraction of core genome, 89.6%) to those of all other *P. gallaeciensis* strains from aquacultures. A genome conservation such as that observed in *Phaeobacter* is rare and even exceeds that of species with specialized ecological niches like the oligotrophic pelagic SAR11 subclade 1a (77.7% fraction of the core genome; Grote et al., 2012) or symbiotic *Vibrio fischeri* (80.4% core genome; Bongrand et al., 2016). Only some small obligate intracellular bacteria like the *Chlamydia psittaci* group (89.5% core genome; Voigt et al., 2012) and *Rickettsia* (84–93% core genome; Fuxelius et al., 2008) reach a similarly high genome conservation, but have a much smaller genome size. This raises the question which mechanisms underlie the unexpected high similarity of the *P. gallaeciensis* genomes.

Based on the homogenous random distribution of SNPs over the genome, recombination events and gene-specific sweeps through the population of *P. gallaeciensis* are unlikely (Marttinen et al., 2012; Shapiro et al., 2012). Instead, the different lineages must have diversified by neutral mutations. The two Pacific *P. gallaeciensis* strains detected in the mesozooplankton fraction differed by only 17 SNPs and were isolated from water samples 1500 km apart. Water currents have been shown to transport Pacific copepods over large distances up to 5000 km (Tatebe et al., 2010). The coastal strains of *P. gallaeciensis* for which genomes are available were isolated from aquacultures in Spain and France (Supplementary Table S1) which are located at a distance of 8,600–10,200 km to the habitats of the Pacific *P. gallaeciensis* isolates. These aquaculture strains differed by 83,953 SNPs from the Pacific strains. Our data allow to infer hypotheses regarding the mechanism of genomic differentiation within the species *P. gallaeciensis*. The rate of spontaneous mutation has recently been determined for *Ruegeria pomeroyi*, another member of the *Roseobacter* group, as 1.39⋅10^-10^ base^-1^ generation^-1^ (Sun et al., 2017). Accordingly, *P. gallaeciensis* with a genome size of 4.54 Mb would take 1585 generations to acquire a SNP. *R. pomeroyi* reaches 45 generations per year in its marine environment (Sun et al., 2017). Due to the higher substrate supply, bacteria associated with marine copepods grow at rates which are 3–18 times higher than free-living bacteria (Tang et al., 2010). They can attain growth rates between 0.7 and 1.2 d^-1^, corresponding to generation times of 0.58–0.99 days, depending on the feeding status of the zooplankton (Tang, 2005; Møller et al., 2007). Based on these faster growth rates, the 1585 generations would take between 919 and 1569 days or 2.5 to 4.3 years. Correspondingly, the 17 SNPs that distinguish the two genomes of the Pacific strains may have accumulated over 20 to 38.7 years. By a similar calculation, it would take between 164,940 and 180,501 years to acquire the larger number of SNPs that distinguish the Pacific from the phylogenetically related aquaculture strains. This time frame is substantially larger than the time required for the global overturn of ocean waters by surface currents and thermohaline circulation (1000–2000 years; Döös et al., 2012) which renders geographic isolation in different water bodies a highly unlikely mechanism of genomic divergence in *P. gallaeciensis*.

## Conclusion

Our data indicate that a previously unknown, distinct clade of *P. gallaeciensis* acquired a limited number of clade-specific genes which may be relevant for its association with mesozooplankton and the colonization of the marine pelagial. The divergence of the Pacific clade was most likely driven by the adaptation to this novel ecological niche rather than by geographic isolation. The distribution pattern observed in the present study also provides first indications for possible biotic interactions between the pelagic lineage of *P. gallaeciensis* and marine mesozooplankton.

## Author Contributions

HF and AM performed the sampling and enrichments. AM conducted the screening and isolation. HF performed the analysis of genome sequences and other data. HF and JO designed the study and wrote the manuscript.

## Conflict of Interest Statement

The authors declare that the research was conducted in the absence of any commercial or financial relationships that could be construed as a potential conflict of interest.

## Footnotes

1 http://ocean-microbiome.embl.de/companion.html

2 https://github.com/boykebunk/genomefinish

## Supplementary Material

FIGURE S1 Flow scheme of combined methods applied in the current study.

FIGURE S2 Distribution of nucleotide diversity (Pi) per gene between *P. gallaeciensis* clades ordered along the genome of P75 by means of the locus_tag number.
