# Supplementary figures and images for: Adaptation of Surface-Associated Bacteria to the Open Ocean: A Genomically Distinct Subpopulation of Phaeobacter gallaeciensis Colonizes Pacific Mesozooplankton
Source: Front Microbiol. 2017 Aug 31;8:1659. doi: 10.3389/fmicb.2017.01659 (PMC5583230; doi:10.3389/fmicb.2017.01659)

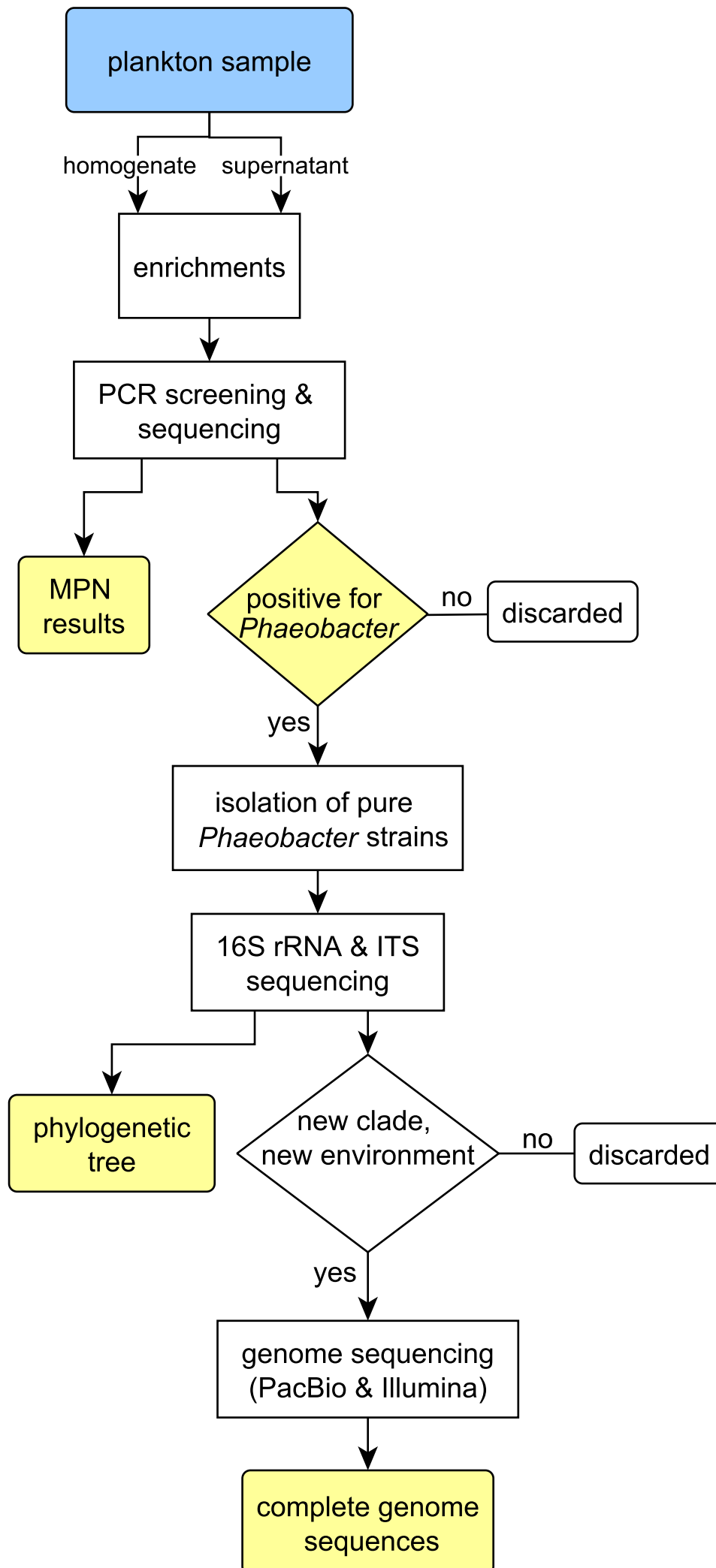

Supplement: FIGURE S1 — Flow scheme of combined methods applied in the current study. [file Image_1.pdf]

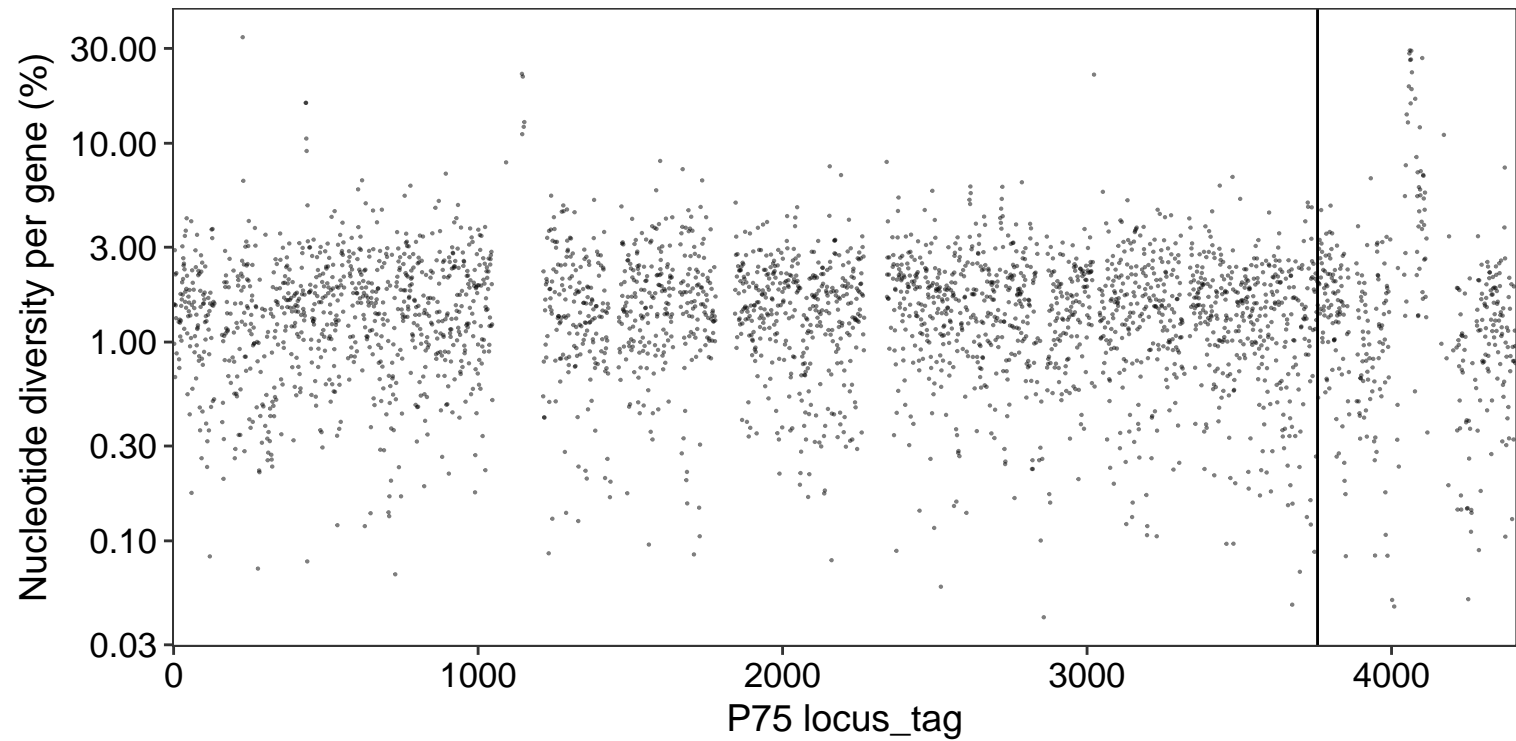

Supplement: FIGURE S2 — Distribution of nucleotide diversity (Pi) per gene between P. gallaeciensis clades ordered along the genome of P75 by means of the locus_tag number. [file Image_2.pdf]
