# Supplementary material for: Adaptation of Surface-Associated Bacteria to the Open Ocean: A Genomically Distinct Subpopulation of Phaeobacter gallaeciensis Colonizes Pacific Mesozooplankton
Source: Front Microbiol. 2017 Aug 31;8:1659. doi: 10.3389/fmicb.2017.01659 (PMC5583230; doi:10.3389/fmicb.2017.01659)
Supplement: Supplementary file 4 [file Data_Sheet_1.DOC]

Supplementary Material

# Adaptation of surface-associated bacteria to the open ocean: A genomically distinct subpopulation of *Phaeobacter gallaeciensis* colonizes Pacific mesozooplankton

Heike M. Freese^*^, Anika Methner, Jörg Overmann

*** Correspondence:** Heike.Freese@dsmz.de

**SUPPLEMENTARY METHODS**

**Screening of cultures**

Cultures in deepwell plates were screened via PCR using the specific forward primer PHA-16S-129f (5’-AAC GTG CCC TTC TCT AAG G-3’; Gram et al., 2015) and the universal reverse primer 907r (5'-CCG TCA ATT CMT TTG AGT TT-3'; Lane, 1991) to detect *Phaeobacter* and closely related genera. Liquid cultures were centrifuged, resuspended in 10 mM Tris (pH 8) and subject to three cycles of heating (96°C) and cooling (15°C). The PCR was carried out with 20 µl reaction mixtures containing 1 μl cell lysate, 300 nM of each primer, 100 μM concentration of each dNTP, 1 mM MgCl_2_, and 0.025 U Hotstar Taq DNA Polymerase (Qiagen) with the corresponding buffer. Cycling conditions comprised an initial denaturation at 95°C for 15 min, 34 cycles at 94°C for 30 sec, 60°C for 30 sec and 72°C for 2.17 min, and a single final extension at 72°C for 10 min. Potential *Phaeobacter* colonies growing on agar plates were detected based on their typical brown color. Partial 16S rRNA gene sequences of all positive liquid cultures and colonies were determined by Sanger sequencing and compared via BLAST (Altschul et al., 1990) against the National Center for Biotechnology Information (NCBI) type material database.

**Genome Sequencing**

For SMRTbell™ template libraries preparation, 8 µg of genomic DNA were sheared (g-tubes™, Covaris, Woburn, MA, USA), the size range monitored by pulse field gel electrophoresis, and DNA fragments end-repaired and ligated to hairpin adapters using the P6 chemistry (Pacific Biosciences, Menlo Park, CA, USA). Illumina libraries were prepared with the Nextera XT DNA Sample Prepararion Kit (lllumina Inc., San Diego, CA, USA) and paired-end sequencing was performed on the MiSeq (PE150) for 75 cycles (yielding ~8 million reads per genome).

**SUPPLEMENTARY FIGURE**

**Supplementary Figure S1.** Flow scheme of combined methods applied in the current study.

**Supplementary Figure S2.** Distribution of nucleotide diversity (Pi) per gene between *P. gallaeciensis* clades ordered along the genome of P75 by means of the locus_tag number.
